# Effects of tillage practice on soil structure, N_2_O emissions and economics in cereal production under current socio-economic conditions in central Bosnia and Herzegovina

**DOI:** 10.1371/journal.pone.0187681

**Published:** 2017-11-08

**Authors:** Ognjen Žurovec, Bishal Kumar Sitaula, Hamid Čustović, Jasminka Žurovec, Peter Dörsch

**Affiliations:** 1 Department of International Environment and Development Studies, Norwegian University of Life Sciences (NMBU), Ås, Norway; 2 Faculty of Agriculture and Food Sciences, University of Sarajevo, Sarajevo, Bosnia and Herzegovina; 3 Faculty of Environmental Sciences and Natural Resource Management, Norwegian University of Life Sciences (NMBU), Ås, Norway; Tennessee State University, UNITED STATES

## Abstract

Conservation tillage is expected to have a positive effect on soil physical properties, soil Carbon (C) storage, while reducing fuel, labour and machinery costs. However, reduced tillage could increase soil nitrous oxide (N_2_O) emissions and offset the expected gains from increased C sequestration. To date, conservation tillage is barely practiced or studied in Bosnia and Herzegovina (BH). Here, we report a field study on the short-term effects of reduced (RT) and no tillage (NT) on N_2_O emission dynamics, yield-scaled N_2_O emissions, soil structure and the economics of cereal production, as compared with conventional tillage (CT). The field experiment was conducted in the Sarajevo region on a clayey loam under typical climatic conditions for humid, continental BH. N_2_O emissions were monitored in a Maize-Barley rotation over two cropping seasons. Soil structure was studied at the end of the second season. In the much wetter 2014, N_2_O emission were in the order of CT > RT > NT, while in the drier 2015, the order was RT > CT > NT. The emission factors were within or slightly above the uncertainty range of the IPCC Tier 1 factor, if taking account for the N input from the cover crop (alfalfa) preceding the first experimental year. Saturated soils in spring, formation of soil crusts and occasional droughts adversely affected yields, particularly in the second year (barley). In 2014, yield-scaled N_2_O emissions ranged from 83.2 to 161.7 g N Mg^-1^ grain (corn) but were much greater in the second year due to crop failure (barley). RT had the smallest yield-scaled N_2_O emission in both years. NT resulted in economically inacceptable returns, due to the increased costs of weed control and low yields in both years. The reduced number of operations in RT reduced production costs and generated positive net returns. Therefore, RT could potentially provide agronomic and environmental benefits in crop production in BH.

## Introduction

It is widely believed that conservation tillage practices such as no-till (NT), minimum or reduced tillage (RT) have beneficial effects on cropping systems relative to conventional tillage (CT). Typically, conservation tillage is associated with improved water infiltration and conservation, reduced erosion and improved soil structure [1, 2] and is perceived as an environmentally sound and sustainable management practice [3]. Recently, conservation tillage practices have been advocated as a measure to mitigate climate change through enhanced soil carbon (C) sequestration [4]. However, C accumulation in soils is finite and the question whether or not agricultural soils lend themselves to sequester relevant amounts of C is currently under debate [5, 6]. One drawback of increased C sequestration into soils may be increased nitrous oxide (N_2_O) emissions, offsetting the “cooling effect” of CO_2_ draw down [7]. Variable effects of NT/RT on N_2_O emissions have been reported [8], varying from decreased to increased N_2_O emissions, especially shortly after shifting from CT to NT [9, 10]. Nevertheless, the benefits of increased soil organic C (SOC) on soil structure, water-nutrient relationships and soil biota are well established [11].

Nitrous oxide (N_2_O) is a greenhouse gas (GHG) with a radiative forcing 298 times that of CO_2_ in a 100-year perspective [12] and currently the most important agent for stratospheric ozone destruction [13]. Agricultural soils are the largest anthropogenic source of N_2_O, associated with the ever-increasing use of synthetic nitrogen (N) and manures [14]. It is estimated that soil-borne N_2_O emissions contribute around 60% to the total anthropogenic climate footprint of agriculture [15]. N_2_O is a product of the microbial N transformations denitrification and nitrifier denitrification in soil, but it is also produced as a by-product during nitrification and during dissimilatory reduction of NO_3_^-^ to NH_4_^+^ [16]. Soil denitrification produces both N_2_O and N_2_, hence this process may serve either as a source or a sink for N_2_O [17]. The rate and product ratio (N_2_O/N_2_) of denitrification in soil depends on various factors such as the amount and availability of mineral N, the C:N ratio of the soil organic matter, the pH as well as temperature and soil moisture content [18]. According to Seitzinger et al. [19], approximately 40% of the 270 Tg N yr ^−1^ globally added to terrestrial ecosystems are removed by soil denitrification.

Soil management can lead to degradation of soil structure [20]. Increase in bulk density due to compaction leads to decreased porosity and changes in pore size distribution, which may give rise to decreased soil aeration, reduced water infiltration, formation of crusts, reduced plant root growth, changes in biological processes and delayed germination and emergence of seedlings [21–23]. Conservation tillage, combined with permanent soil cover, has been shown to result in a build-up of SOC in soil surface layers and has a potential to improve soil structure and to increase infiltration of water, thus reducing water runoff and erosion [20, 24].

Minimum soil disturbance is one of its three main pillars of conservation agriculture [3]. So far, conservation agriculture has been adopted mainly in countries in which highly mechanized and high-input agricultural production prevails [25]. The reason for this is CA’s documented ability to reduce land degradation, soil erosion and to reduce fuel costs [26]. Implementing CA in a country with prevailing low-input smallholder agriculture, such as Bosnia and Herzegovina (BH), could thus have a potential to stabilise crop yields and improve soil conditions, while being less labour intensive and more cost-efficient, especially in rain-fed agriculture. However, a number of obstacles challenges implementation of CA in BH. Agriculture in BH today is characterized by smallholder farmers with low financial capital and high risk aversion, pursuing subsistence farming in mixed crop/livestock systems on limited land resources [27]. This has resulted in decreased productivity due to inadequate technical equipment and mechanisation and lack of education, similar to what has been described for developing countries [28]. Smallholders have become risk averse in applying costly inputs, such as fertilizers and pesticides, which has resulted in significant yield reductions. In addition, the existence of counterfeits and low quality products, such as seed material, fertilizers and pesticides, reduces trust in the effectiveness of agricultural inputs [29]. Due to the lack of investment in modern agricultural machinery, smallholder farmers in BH and Western Balkan are forced to apply conventional tilling methods using existing machinery with small working width, which increases the production costs due to the increased number of passages and increased fuel consumption [30].

First attempts to investigate the benefits of conservation tillage in the Western Balkan region were made in former Yugoslavia in the 1960ies and 70ies [31]. Yet, conservation tillage is very little applied to date. Despite a renewed interest in tillage research in BH and the neighbouring countries in the past decade [32, 33], long-term effects of alternative tillage methods on soil structure, crop yields and N_2_O emissions under the current agro-ecological conditions are largely unknown. In fact, while much is known about the impact of fertilizer N on different agricultural crops, N_2_O emissions and its underlying variables have never been studied in BH or the western Balkan region.

The objectives of the present study were i) to examine the effects of CT, RT and NT on soil structure, crop yields and N_2_O emissions, ii) to compare yield-scaled emissions in the three tillage systems and iii) to evaluate the economics of RT and NT with respect to the likelihood of its adoption by small-scale farmers under the current socio-economic conditions. For this, we set up a field experiment under typical pedo- climatic conditions of continental BH and conducted weekly N_2_O measurements in a Maize-Barley rotation over two growing seasons. Soil structure was measured at the end of the second year.

## Materials and methods

### Experimental site

This study was carried out on the research farm Butmir of the Faculty of Agricultural and Food Sciences in Sarajevo, Bosnia and Herzegovina (43°49'N, 18°19'E, 547 m a.s.l.) from December 2013 to December 2015. The study site has a continental humid climate with a mean annual temperature of 9.6 ^o^C and a mean annual precipitation of 899 mm. The soil is classified as a Fluvisol, with a pH of 6.40 and a total C and N content of 1.34% and 0.14%, respectively. The texture class is a clayey loam, with 41.5, 24.6 and 33.9% of sand, silt and clay, respectively. The experiment included three tillage treatments laid out in a strip design with four subplots per treatment.

- Conventional tillage (CT): autumn ploughing to 30 cm depth and secondary tillage with a roto-tiller in spring to 15 cm depth before seedbed preparation
- Reduced tillage (RT): no autumn ploughing but disking to 15 cm depth in spring before seedbed preparation
- No tillage (NT): direct sowing into the untilled soil. Since no specialised NT seed drill was available, we used a traditional mechanical drill with an added ballast for increased penetration strength

The treatments were established in three 50 m long and 5 m wide stripes. Each strip was divided in four 50 m^2^ subplots for GHG exchange and yield measurements. The crop rotation consisted of Corn (*Zea mays L*.) in 2014 and spring Barley (*Hordeum vulgare L*.) in 2015. At the time establishing the experiment in autumn 2013, the field was uniformly grown to Alfalfa, which was either autumn ploughed (CT) in 2013 and tilled in spring 2014, chemically mulched and partly incorporated by disking (RT) in spring 2014, or mulched entirely chemically in spring 2014 (NT). Crop residues in 2014 (corn) were milled with a silo combine and fully (CT) or partially (RT) incorporated into soil during autumn ploughing (CT) and disking (RT), or used as a mulch (NT). The same fertilizer rate was used for all treatments. We used N fertilizers which are common and accessible on the local market and chose fertilization rates which resemble those commonly used by smallholder farmers in BH. N fertilization was carried out by mechanical spreading of 250 kg ha^-1^ CAN (Calcium-Ammonium-Nitrate) in July 2014, one month after sowing, and 450 kg ha^-1^ NPK 15:15:15 applied in April 2015, during seedbed preparation. Both rates of fertilizer applied are equivalent to a fertilization rate of 67.5 kg N ha^-1^.

Weed control was carried out with glyphosate in NT and RT (before seedbed preparation) in both years. Broad leaf and grass-weed control in the first year was carried out using 2,4-Dichlorophenoxyacetic acid after sowing and pre-emergence, and the combination of Nicosulfuron and Prosulfuron in post-emergence. Broad leaf weeds in barley were controlled with 2,4-Dichlorophenoxyacetic acid in post-emergence in the second year. The experiment was carried out under rain-fed conditions (only 0.4% of BH’s arable land is irrigated [27]).

Monthly and yearly meteorological data for the period 1961–2010 and the daily data for the study period were obtained from the meteorological station at Sarajevo International Airport (43°49’N, 18°20’E) situated close to experimental site and provided by the Federal Hydrometeorological Institute of Bosnia and Herzegovina.

### Field fluxes of N_2_O and the derived emission factor and intensity

Weekly to bi-weekly measurements of N_2_O emissions were carried out from December 2013 to December 2014 and from March 2015 to December 2015. On each sampling date, fluxes were measured between 4 and 6 pm in the afternoon, in an attempt to circumvent the bias arising from randomly sampling diurnal variation in N_2_O emission. Both midday and night-time maxima have been reported [34]. It is noteworthy, however, that fluxes were measured within one hour in all treatments, so that differences between treatments should not be due to diurnal variation. The fluxes were measured following the methodology described by Nadeem et al. [35], using static aluminium chambers. Aluminium frames (60×60×15 cm) were permanently installed in the field and only removed for field operations and placed back at the same location in the plot. A total of 12 frames were installed on the experimental field, giving four replicates plots for each treatment (CT, RT, NT). Gas sampling was carried out by deploying the chambers (62×62×30 cm) on the frames for 45 min and withdrawing 15-ml gas samples from the chamber headspace using a 20-ml polypropylene syringe with stopcock at regular intervals of 15 min (0, 15, 30 and 45 min). The samples (15 ml) were transferred to pre-evacuated 12-ml glass vials crimped with butyl rubber septa resulting in an overpressure in the vials to avoid contamination during sample storage. Flux measurements were carried out every 7–10 days throughout the entire research period and every 3–5 days in the month after fertilization in 2015. The gas samples were analysed at the Norwegian University of Life Sciences, using a gas chromatograph (GC, Model 7890 A, Agilant, Santa Clara, CA, USA) equipped with a 30-m-wide bore Poraplot Q (0.53 mm) column run at 38°C with back flushing and helium (He) as a carrier gas. The electron capture detector (ECD) was run at 375°C with 17 ml min^−1^ ArCH4 (90/10 Vol %) as makeup gas. The GC was connected to an autosampler via a peristaltic pump (Gilson minipuls 3, Middleton, W1, USA) pumping approximately 2.5 ml gas through a 250-μl sampling loop maintained at 1 Atm pressure. The injection system was back-flushed by helium (6.0) before each sampling to minimize memory effects. Temperatures inside the chamber and above the soil surface were used to calculate an average temperature during flux sampling. Rates of N_2_O emission were estimated by fitting either a linear (R^2^ ≥ 0.85) or a quadratic function to the observed N_2_O accumulation over time. For this, all fluxes were inspected graphically and fluxes with a R^2^ < 0.85 and a flux density of < 5 mg N m^-2^ h^-1^ were set to zero. N_2_O flux was calculated according to Eq 1:

$$F_{N2O}=\frac{d_{N2O}}{dt}\times \frac{V_{c}}{A}\times \frac{M_{N}}{V_{m}}\times 60$$ (1)

where *F*_*N2O*_ is the N_2_O emission flux (μg N_2_O-N m^−2^ h^−1^), *d*_*N2O*_*/dt* is the relative change in N_2_O concentration in the chamber headspace (ppmv min^−1^), *Vc* is the chamber volume (L), *A* is the area covered by the chamber (m^2^), *M*_*N*_ is the molecular mass of N in N_2_O (g mol^−1^) and *Vm* is the molecular volume of gas at chamber temperature (L mol^−1^).

Cumulative fluxes were calculated by plotting daily average N_2_O fluxes against time, interpolating linearly between them, and integrating the area under the curve [36]. Cumulative flux in 2014 represents area-scaled emissions for the entire year, while in 2015 it represents the cumulative flux for 226 days of the year (flux measurements started on 20^th^ of March) plus linearly interpolated values for the missing period, assuming small off-season emissions like observed in the previous year. Emission factors were calculated as the fraction of applied fertilizer N emitted as N_2_O-N, assuming background N_2_O emission of 1 kg N ha^−1^ year^−1^ [37]. Yield-scaled N_2_O emission was calculated as emission intensity, which is a function of N fertilization rate and expressed as N_2_O-N (g) emitted per ton of grain yield [38]. To account for the N-input by preceding alfalfa in the first cropping year, we estimated the N returned to soil with the crowns and roots based in literature values. According to Kelner et al. [39], this amount is 107 kg N ha^-1^ for a 1-year stand of alfalfa. However, when the yields of the first cut from that study are compared with results reported by Junuzović [40] for our field, we reduced the N yield by roughly 50% to a more realistic N input of 53.5 kg N ha^-1^.

### Soil measurements

Soil moisture and temperature at 5 cm depth were measured daily using data loggers (Decagon EM50, Pullman, WA, USA) together with ECH2O sensors (Decagon) for volumetric soil water content (VSWC) and temperature in three replicates per treatment. No measurements are available for the period between 2 April and 24 June 2014, due to equipment failure.

Soil physical properties were analysed in undisturbed soil cores (100 cm^3^ stainless steel cylinders) collected in July 2015 in four replicates per treatment. Particle density (PD) was determined using an air pycnometer according to Langer. Bulk density (PD) was determined gravimetrically. BD and PD were used to calculate total porosity, which was used to convert VSWC to water filled pore space (WFPS) by Eq 2:

$$WFPS=\frac{VSWC}{1-\frac{BD}{PD}}$$ (2)

Soil water retention curves between pF 1.8 and 4.2 were determined using ceramic pressure plate extractors [41]. These results were then used to determine soil pore size distributions in different tillage treatments.

In 2015, soil samples were taken from 0 to 15 cm depth at each gas sampling date. Multiple cores from each treatment (4 per subplot) were homogenized, bulked and frozen. Immediately after thawing, 45 g fresh weight soil was extracted by 30 minutes of horizontal shaking in a 50 ml 2M KCl solution. The extracts were filtered and soil mineral N (NH_4_^+^, NO_3_^−^) was analysed by colorimetry as described by Keeney [42].

### Yield and economic parameters

Yields were measured on each subplot as dry grain corn in 2014 and barley in 2015 standardized to 14% moisture content. Production costs were estimated for each of the three tillage systems. Input items such as seed, fertilizer and chemicals applied were purchased from local retailers and the exact prices were recorded. Average purchase market prices for the crops used in the experiment are taken from Agency for Statistics of Bosnia and Herzegovina [43, 44] for the respective years. For an approximation of labour and tillage operation costs, we used data from the local agricultural extension service, which were cross-validated with farmers in the same area. The results were calculated and discussed as the difference in net return per hectare in the three tillage systems, which was calculated from net income for crop after deducting all variable costs. We refrained to estimate fixed costs for production systems in this study due to high variability in possession of tractors and other necessary mechanisation and assets, which often have a long depreciation life and the general tendency to become obsolete [45].

### Statistical analysis

N_2_O emission rates were log-transformed to approach normal Gaussian distribution. To test the effects of treatment and year, repeated measures two-way ANOVA followed by Bonferroni post-test was performed for N_2_O flux data, soil temperature and WFPS. One-way ANOVA followed by a Tukey’s multiple comparison post test was performed for soil NH_4_^+^ and NO_3_^−^, cumulative N_2_O emissions and yield-scaled N_2_O emissions. All data were analysed using SPSS ver. 24 (IBM Corp, USA).

## Results

### Weather conditions

**Fig 1:**
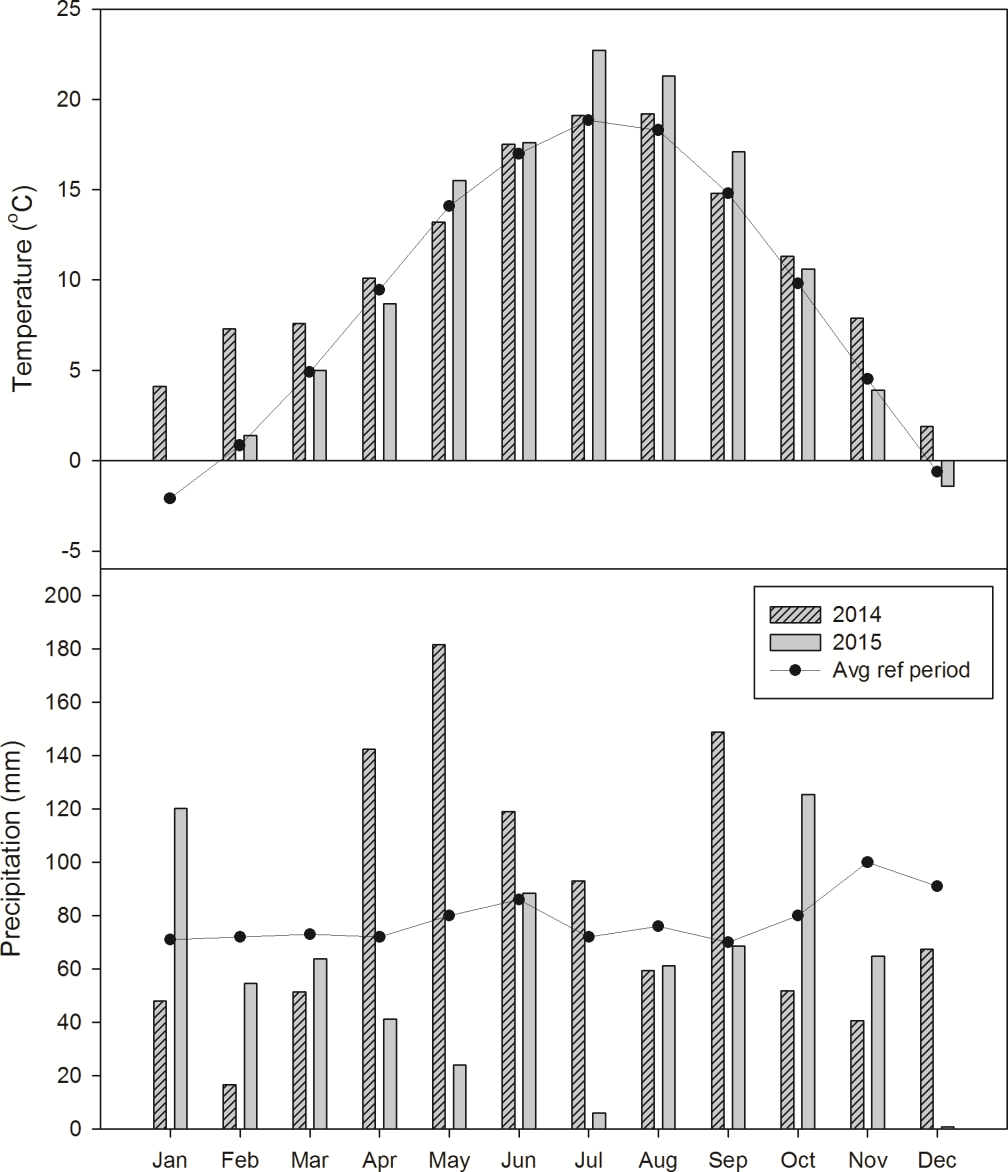
Average monthly temperatures and precipitation close to the sampling site.

The average temperature in 2014 and 2015 was 11.2 and 10.2 ^o^C, respectively, which is warmer than the long-term reference temperature (9.6 ^o^C). The year 2014 had an exceptionally warm winter (Jan-March, 2015, Fig 1), while monthly temperatures for the rest of the year were similar to the long-term average values. Monthly average temperatures in summer 2015 were clearly higher than the reference temperatures. (Fig 1).

Both the amount of annual precipitation and the seasonal distribution varied greatly between the two years. Cumulative precipitation was larger (1020 mm) in 2014, while it was smaller in 2015 (719 mm) compared with the reference precipitation (899 mm). The annual rain distribution in 2014 showed excessive amounts of precipitation in April, May and September and the amount of precipitation was larger than the monthly reference precipitation throughout most of the cropping season. By contrast, 2015 was a dry year, with extremely low precipitation in the cropping season, especially May and July, with monthly precipitation sums of only 24 and 6 mm, respectively.

### N_2_O emission dynamics and ancillary variables

**Fig 2:**
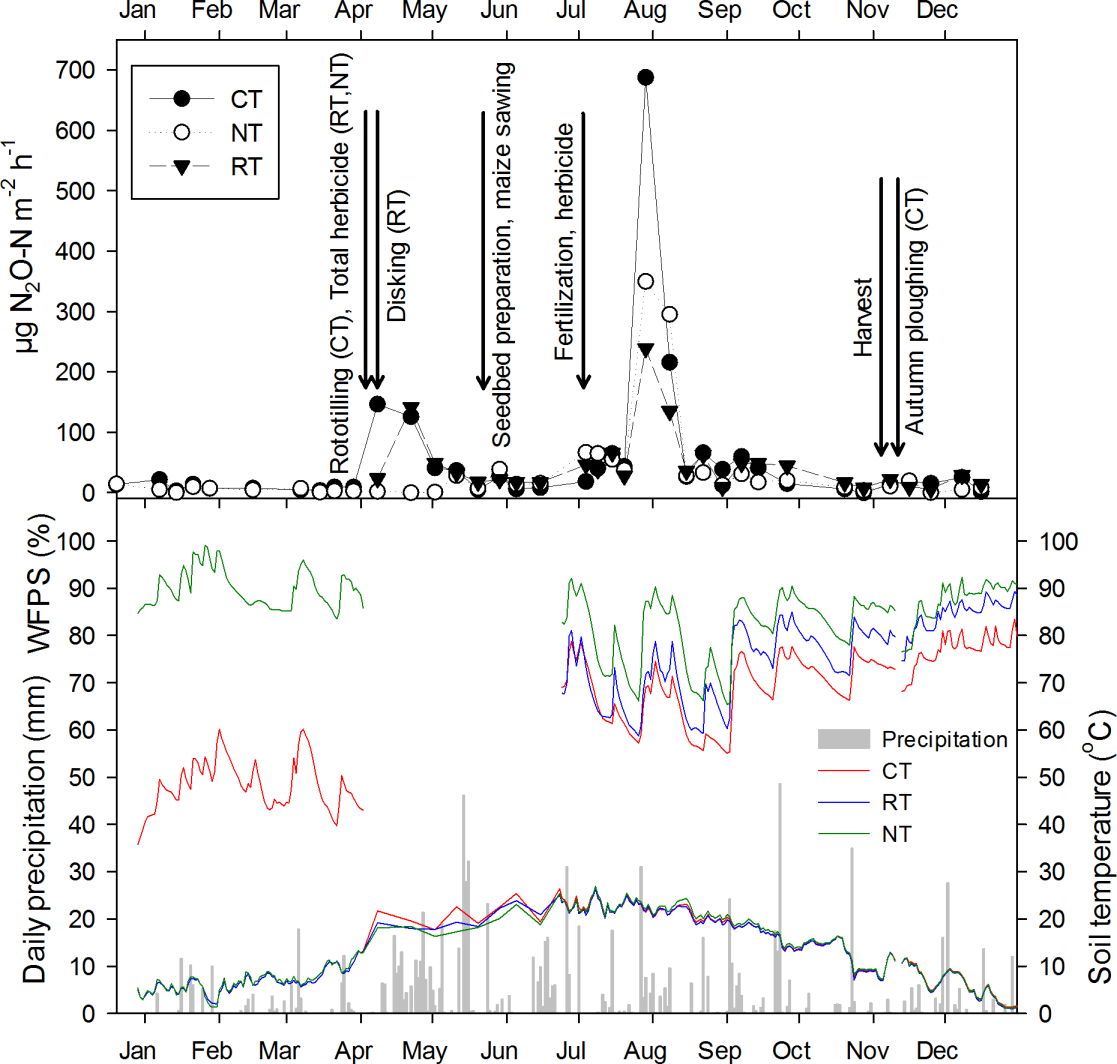
**a** Mean N_2_O emissions per treatment (n = 4) and **b** daily precipitation, and mean soil temperature and moisture in 5 cm depth in CT, NT and RT treatments in 2014. Error bars are omitted to maintain readability.

**Fig 3:**
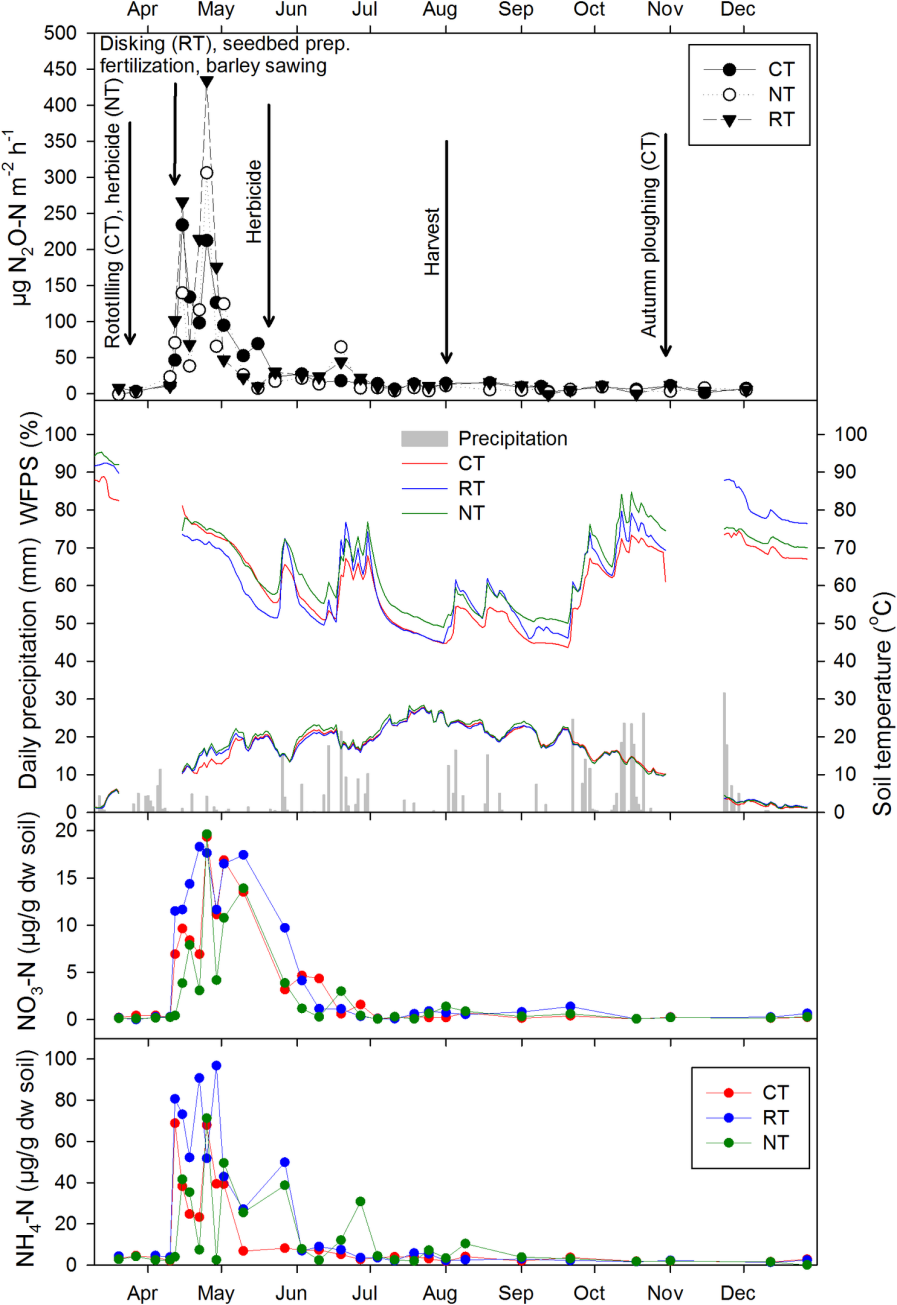
**a** Mean N_2_O emissions (n = 4), **b** daily precipitation, mean soil temperature and moisture in 5 cm depth, **c** Soil NO_3_^-^ and **d** soil NH_4_^+^ concentrations in 0–15 cm depth CT, NT and RT treatments in 2015. Error bars are omitted to maintain readability.

All treatments had similar N_2_O emission dynamics, although peak values differed during certain periods, most notably after fertilization (Figs 2A and 3A). Two-way repeated measures ANOVA showed that tillage and experimental year had a significant effect on N_2_O flux (P < 0.05), while the interaction between tillage and experimental year was not significant. According to Bonferroni pairwise comparison, the difference in N_2_O emissions between CT and NT was significant only for the 90% confidence interval in the first year, while there was a significant difference between RT and NT (P < 0.05) in the second year. All peak emissions coincided with high WFPS values and elevated soil temperatures (Figs 2B and 3B). In contrast, winter emissions in 2014 (January–April) were small, despite large WFPS values in non-tilled soil. The first N_2_O emission peak occurred at the beginning of April, at the time of rototillage in CT; Emission rates in CT and RT increased, whereas they remained small in NT. The largest N_2_O emission rates were recorded, regardless of treatment, in August 2014, one month after fertilization, when soil moisture started to fluctuate (Fig 2A and 2B). Maximum observed fluxes (treatment means per date) were 587.1 μg N_2_O-N m^-2^ h^-1^ for CT, 373.7 for NT and 301.0 for RT. Emission rates remained slightly elevated for the remainder of the cropping period in all treatments, before decreasing gradually with decreasing soil temperature to low background values one month before harvest (first week of November).

In 2015, N_2_O measurements started right before tillage operations in March (Fig 3A). Emission rates remained small during seedbed preparation and herbicide treatment until fertilization and sowing of barley in the beginning of April. N_2_O emission rates increased strongly in all treatments immediately after fertilization and remained large throughout April, while the soil was still wet (>60% WFPS; Fig 3B). Maximum emission rates were measured three weeks after fertilization, with treatment means of 434.4 μg N_2_O-N m^-2^ h^-1^ for RT, 306.2 for NT and 234.0 μg N_2_O-N m^-2^ h^-1^ for CT. When WFPS values dropped below 60% in the middle of May, N_2_O emission rates receded. A smaller, transient emission peak was observed in NT and RT, but not in CT plots, in the middle of June, which coincided with rewetting of dry soil by rain. Pronounced drying-rewetting later in 2015 did not induce measurable N_2_O emission response, nor did harvest and ploughing, which is in line with small mineral N concentrations during this period (Fig 3C and 3D).

To find out whether tillage treatments had any effect on known drivers for N_2_O emission, we tested for differences in soil temperatures and WFPS in both years and for differences in extractable NH_4_^+^ or NO_3_^−^ in 2015 across treatments. Mean daily soil temperature showed little difference between treatments (Figs 2B and 3B), but annual average soil temperature was slightly higher in NT plots in both years (12.8 and 13.2 ^o^C) than in CT (12.6 and 12.8) and RT plots (12.5 and 12.8) and statistically significant (P < 0.001).

In both years, CT soils had consistently smaller WFPS values than RT or NT soil. This contrast was most pronounced after ploughing (CT) and secondary tillage (CT and RT). During the wet summer of 2014, WFPS values of NT soil were almost constantly within the range of 80–90% WFPS, often exceeding 90%. On average, WFPS of NT soil exceeded that of CT and RT soil by 22.1% and 4.6%, respectively. Also in the dry summer of 2015, NT soil had the highest average WFPS values, although the difference between the treatments was less pronounced than in 2014 (6.4% and 3.7% higher than in CT and RT soils, respectively). Both tillage, experimental year and their interaction had a highly significant effect on WFPS and the difference was significant between every treatment (P < 0.0001).

No significant difference between treatments was found for extractable NH_4_^+^ or NO_3_^−^ at 0–15 cm depth in 2015. The mineral N content increased rapidly after fertilization (Fig 3C and 3D), before levelling off to low background values by the end of June. The increase in NH_4_^+^ was somewhat delayed in NT soil, reflecting the difference between surface applied (NT) and incorporated (during seedbed preparation in CT and RT) fertilizer. Concentrations of NO_3_^−^ declined to below 10 μg g^-1^ one month after fertilization and remained at very low levels for the remainder of the growing season. Similar to NO_3_^−^, NH_4_^+^ concentrations declined to values lower than 10 μg g dw soil^-1^ two months after fertilization and remained low until the end of the year. The only difference was that NT showed occasionally elevated concentrations of NH_4_^+^, likely reflecting drying-rewetting induced mineralization pulses.

### Soil properties and crop yields

By August 2015, i.e. in the second experimental year, two months after seedbed preparation, tillage regime had resulted in clear differences in soil bulk density, total porosity and pore size distribution (Table 1). CT soil had the smallest bulk density among all treatments, which was significantly different from that of NT (P < 0.05). Accordingly, CT soil had a larger porosity and a larger share of medium and macro pores. Increased bulk density in NT combined with reduced infiltration resulted in soil crusting and occasional waterlogging during both growing seasons.

**Table 1:** Soil physical properties 20 months after the establishment of contrasting tillage regimes (August 2015).

Treatment	Bulk density* (g cm^-3^)	Total porosity (%)	Pore size distribution (%)		
			Micro (<0.2 μm)	Meso (0.2–10 μm)	Macro (>10 μm)
CT	1.36 (±0.03)^a^	53.56^a^	13.85^a^	27.03^a^	12.68^a^
RT	1.42 (±0.04)	51.07^a^	12.57^a^	26.53^a^	11.97^a^
NT	1.55 (±0.04)^b^	45.40^b^	15.72^b^	21.47^b^	8.22^b^

* SE shown in brackets

^a,b^ Different letters indicate significant differences across treatments (Bonferroni, P < 0.05)

Next to soil properties, weather conditions had a major impact on crop yields in both cropping seasons. Excessive rainfall during spring 2014 delayed most of agricultural operations, including rototilling, disking, seedbed preparation and sowing of corn for more than one month, which led to late harvest and yield decrease. The same weather conditions occurred in the following year, resulting in late sowing of barley. This delay, coupled with two drought periods in May and July, led to crop failure. Average crop yields per treatment and year are shown in Table 2.

**Table 2:** Average crop yields corrected for 14% moisture in CT, RT and NT.

Year—crop	Treatment	Average yield (kg ha^-1^) ± SD (n = 4)
	CT	6561.9 ± 678.7^a^
2014—corn	RT	6165.8 ± 790.3^a^
	NT	4314.2 ± 1118.2^b^
	CT	1508.1 ± 139.7^a^
2015—barley	RT	2185.0 ± 254.3^b^
	NT	1571.5 ± 135.2^a^

^a,b^ Different letters indicate significant differences across treatments (Bonferroni, P < 0.05)

### Cumulative N_2_O emissions and emission factors

In 2014, cumulative N_2_O emission was largest in CT and significantly different from RT and NT only at P < 0.1 (Table 3). Cumulative N_2_O emissions in 2015 were significantly smaller (P < 0.005) than in 2014 for all treatments and RT had the highest emission, followed by CT and NT, with the difference between RT and NT treatments being significant (P < 0.05).

**Table 3:** Cumulative N_2_O emissions.

Season	Cumulative N_2_O emission (kg N_2_O-N ha^-1^)*		
	CT	RT	NT
2014	4.3 (±0.6)^a^	3.0 (±0.2)^b^	2.8 (±0.4)^b^
2015	1.7 (±0.1)	1.9 (±0.1)^a^	1.4 (±0.05)^b^

* SE shown in brackets

^a,b^ Different letters indicate significant differences across treatments (Bonferroni, P < 0.1 in 2014 and P < 0.05 in 2015)

**Fig 4:**
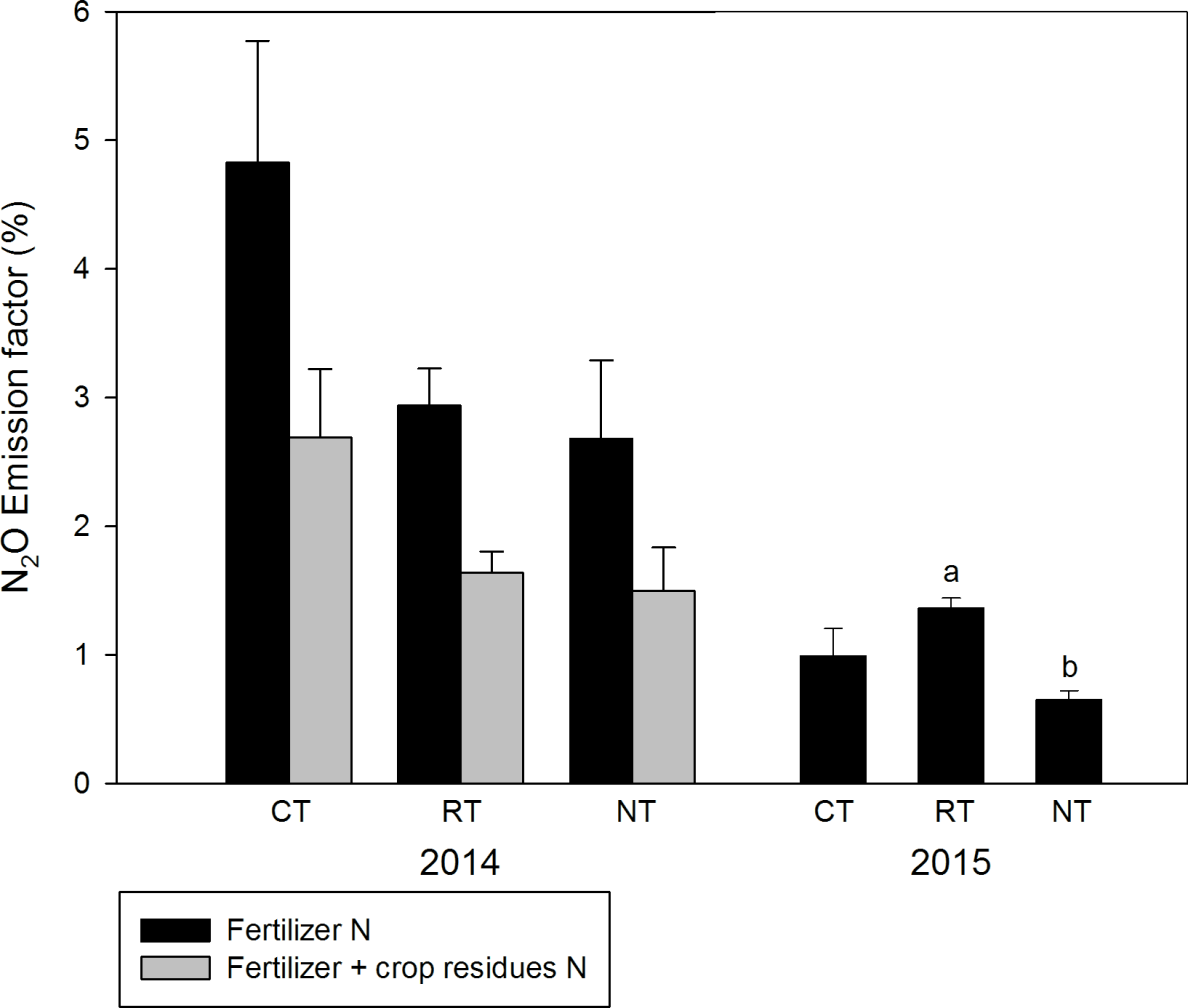
N_2_O emission factors. Different letters indicate significant differences across treatments at P < 0.05.

Annual N_2_O emission factors, assuming a background emission of 1 kg N_2_O-N ha^-1^ y^-1^ [37], are shown in Fig 4. Extraordinary large apparent emission factors were found in 2014 with 4.8% for CT, 2.9% for RT and 2.6% for NT, which likely reflect the extra N from alfalfa incorporated in autumn 2013 (CT) and spring 2014 (RT), or mulched in spring 2014 (RT, NT). We therefore estimated the amount of N from alfalfa residues based on N content and crown-root ratios reported in the literature as well as measured yields and added this N to the fertilizer N. Estimated N input by alfalfa crop residues was 53.5 kg N ha^-1^ for all three treatments. This resulted in more realistic emission factors of 1.5, 1.6 and 2.7% for NT, RT and CT, respectively, in the year of establishing the three tillage treatments. Irrespective of the amount of N input estimated, CT hat the largest emission factor in 2014. Emission factors in the dry year 2015 were much smaller and in the order of 0.7, 1.0 and 1.5% for NT, CT and RT, respectively.

**Fig 5:**
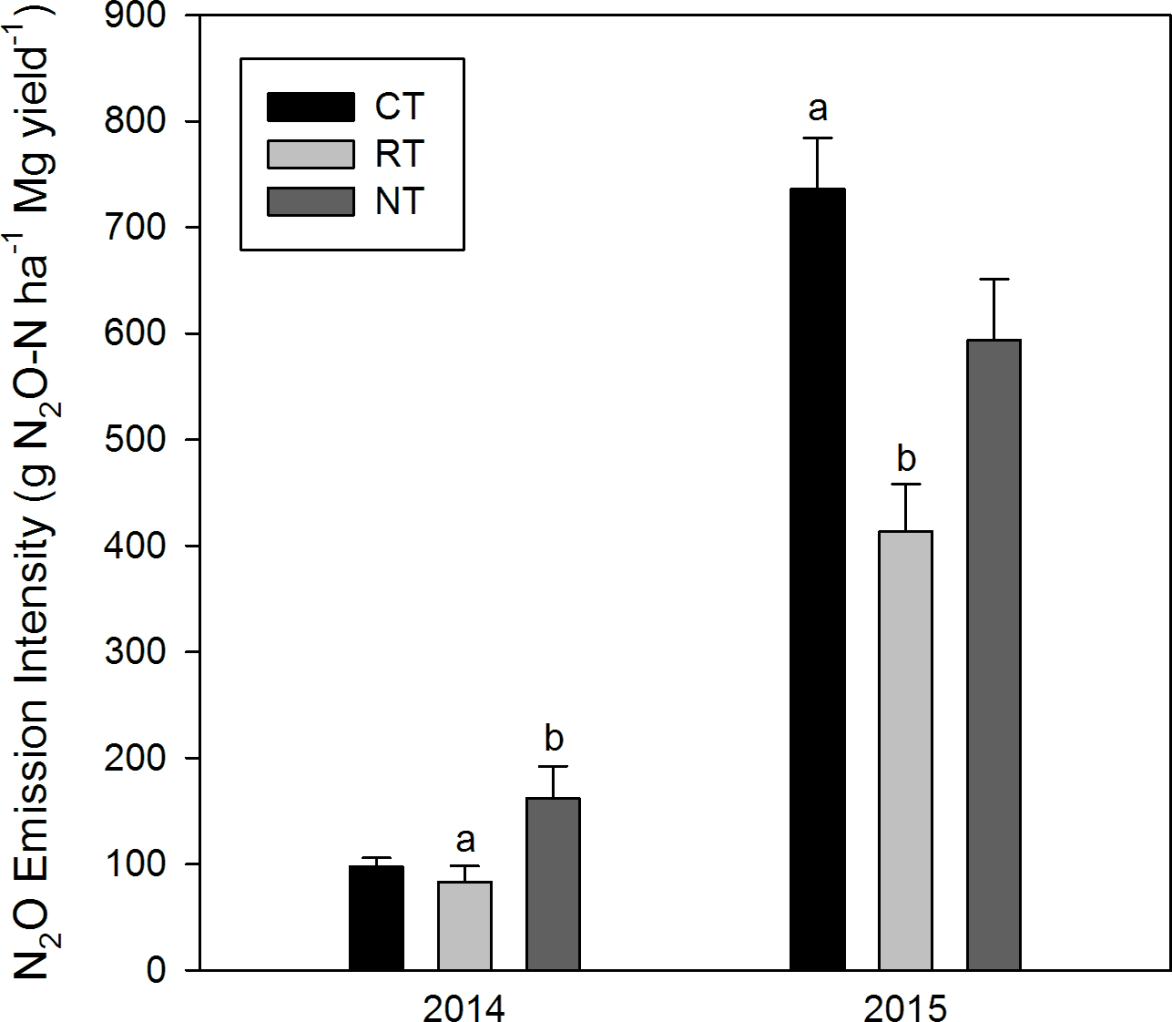
Yield-scaled N_2_O emissions. Different letters indicate significant differences across treatments at P < 0.05.

Yield-scaled N_2_O emission (Fig 5) was smallest for RT plots in both cropping seasons, ranging from 83.2 (2014) to 413.5 g N_2_O-N Mg^-1^ (2015). NT had the largest yield-scaled N_2_O emission in 2014 (161.7), while it was largest for CT in 2015 (736.1). Very high values in 2015 are the result of crop failure caused by drought. Analysis by one-way ANOVA followed by a Tukey’s multiple comparison post-test showed statistically significant differences between RT and NT (P < 0.05) in 2014 and between CT and RT (P < 0.005) in 2015

### Economic comparison between tillage methods

Based on estimated net return per hectare in the different tillage systems (Table 4), it is evident that the reduced number of cultivation steps in NT decreased production costs as compared to CT. However, this reduction was zeroed by increased costs for weed control. With an overall smaller yield in NT, this resulted in markedly smaller net return in NT than in RT or CT in 2014. There was a notable difference in net return between the two years among the tillage treatments. The differences in yield between the two cropping seasons can be mainly attributed to different weather conditions, which lead to yield reduction in the first year and crop failure in the second year. Notably, RT had large net return, close to that of CT, in the first year and the least negative net return in the second year. CT had largest net return in the first year and smallest in the second year. NT achieved a small net return in the first year in comparison to CT and RT, while it had less negative net return than CT in the second year.

**Table 4:** Net income per hectare under different tillage systems based on the difference of crop net income and variable costs of production.

Season/Crop	Price (BAM)*		
**2014 Corn**	**CT**	**RT**	**NT**
Seed cost	240	240	240
Fertilizer and application	265	265	265
Tillage operations, fuel, maintenance, labour	272	146	80
Herbicide and application	99	188	233
Harvesting (hired machinery and labour)	300	300	300
*Total variable costs*	1176	1139	1118
	Yield (t)		
	6.56	6.17	4.31
	Price (BAM per t)		268.4
*Net income*	1761.3	1655.0	1158.0
**Net return**	**585.3**	**516.0**	**40.0**
**2015 Barley**	**CT**	**RT**	**NT**
Seed cost	200	200	200
Fertilizer and application	475	475	475
Tillage, fuel, maintenance, labour	265	142	70
Herbicide and application	54	143	188
Harvesting (hired machinery and labour)	300	300	300
*Total variable costs*	1294	1260	1233
	Yield (t)		
	1.51	2.18	1.57
	Price (BAM per t)		388.63
*Net income*	586.1	849.1	610.7
**Net return**	**-707.9**	**-410.9**	**-622.3**

* 1 BAM = 0.51 EUR

## Discussion

### Tillage effects on N_2_O fluxes and soil variables

N_2_O emissions showed a similar N fertilization response in all treatments and consequently similar N_2_O emission rates in both years. Between 35% (NT) and 57% (CT) of the cumulative annual N_2_O emission occurred during the first month after fertilization in 2014, while this share was between 54 and 57% in 2015. In each of the two growing seasons, temporal N_2_O emission patterns resembled each other irrespective of treatment, with only a few notable exceptions. A clear treatment effect was noted after spring tillage operations in 2014, when N_2_O emissions increased in CT and RT plots, but not in NT plots. While elevated emissions in CT and RT may be partly attributed to increased organic matter decomposition triggered by secondary tillage [46], it is important to note that N_2_O emissions in CT did not respond to ploughing in autumn later the same year (Fig 1). This suggests that tillage as such does not induce elevated N_2_O emissions. Increased emissions in RT and CT in April-May 2014 coincided with an extraordinary wet period and increasing soil temperatures (Fig 1B), while the main difference between treatments was that alfalfa residues had been incorporated freshly (RT) or in previous autumn (CT), whereas residues remained undisturbed in NT after chemical mulching, when the peaks in CT and RT occurred. It is likely that the incorporation of the N-rich residues into the wet soil created conditions favourable for mineralisation of C and N from the residues, fuelling N_2_O emissions by nitrification and denitrification. By contrast, chemical mulching of alfalfa in NT created no distinguishable emission peak in 2014. An increase in N_2_O emissions after incorporation of cover crop residues in comparison with residues left on the surface was previously reported in the meta-study by Basche et al. [47]. Alternatively, the first observed emission peak in CT was due to the release of previously produced N_2_O that was trapped in deeper soil pores. However, this effect did not occur shortly after disking in RT. Transiently elevated N_2_O emissions in RT and NT relative to CT were observed on one day in June 2015 (Fig 3A), shortly after abundant rain had increased WFPS values from ~50 to ~75% (Fig 3B), while there was still available NO_3_^-^ present in the soil (Fig 3C). Enhanced N_2_O emissions triggered by drying-rewetting are well documented [48, 49] and are most likely due to denitrification. Interestingly, WFPS increase in CT was smaller than in RT and NT, probably explaining the lack of N_2_O emission response in this treatment. Periods of repeated drought and rewetting later in 2015 did not induce measurable N_2_O emissions, apparently because small concentrations of NH_4_^+^ and NO_3_^−^ limited nitrification and denitrification activity. All peak emissions outside the period directly affected by fertilization coincided with large WFPS (70–90%) and high soil temperatures (>10°C), illustrating the importance of crop residue management (April 2014) and tillage regime (June 2015) for N_2_O emissions outside the period directly affected by fertilization. The greatest differences between treatment means of N_2_O emissions were seen after fertilizer application. In 2014, post-fertilization emissions were greatest in CT, followed by NT and RT (Fig 2A), despite lowest WFPS values in CT (Fig 2B). The likely reason for this is that incorporation of the preceding crop (alfalfa) in autumn 2013 had resulted in a more active microbial community in CT than in RT and NT, which was further stimulated by rototilling in spring 2014. Thus, CT plots responded stronger to N addition than RT or NT plots. In 2015, no N-rich residues were present, and RT responded strongest to N-fertilization (Fig 3A), providing the most favourable conditions for nitrification and denitrification in terms of soil structure and substrate availability.

Despite the relatively short periods of distinct N_2_O flux, we found differences in annual emissions between treatments (Table 3). Since annual emissions were dominated by post-fertilization fluxes, largest annual emissions were found in CT in 2014 and in RT in 2015. NT had the smallest emission in both years, although this difference was not significant in 2014. A meta-analysis of reported N_2_O emissions in 239 paired field trials with conventional tillage (CT) and NT/RT showed no consistent short-term (≤10 years) tillage effect on area-scaled N_2_O emissions when land was converted from CT to NT/RT [8]. Smaller N_2_O emissions from NT soils in a newly established tillage experiment, like the case in our study, were previously reported by Chatskikh and Olesen [50] in a loamy sand soil with barley as a crop, while long-term reduction in N_2_O emission by NT on loams are reported by Gregorich et al. [51] and Mosier et al. [38] for corn-soybean crop rotation. By contrast, larger N_2_O emissions in newly established NT systems than in CT have been reported by Ball et al. [52] and Baggs et al. [53] on loamy soils in humid areas. This indicates that tillage effects on N_2_O emissions are highly variable, both spatially and temporally, and influenced by a wide spectrum of biotic and abiotic factors and their interactions in the specific region. It should be also mentioned, that the sampling frequency used in our study was low and that contrasts in annual N_2_O emission probably underestimated treatment effects, since the resolution was not high enough to capture every N_2_O emission peak.

In our experiment, tillage regime had a clear effect on every measured soil variable. Soil temperatures in NT were on average 0.3–0.4 ^o^C higher and had up to 22.1% larger average WPFS values as compared with CT. Lower bulk density and more medium and macro pores in CT suggested that the ploughed soil was better aerated than RT or NT. Overall, warmer, wetter and less aerated soil in NT would be expected to favour denitrification and hence increased N_2_O emissions, especially in case of heavy soils in humid climates [9]. Interestingly, this effect was not found in our study, which may have a number of management-specific reasons. Firstly, while no cover crops were planned in our study, all fields were uniformly cropped to alfalfa, an N-fixing legume, in the year prior to establishing the experiment. Using alfalfa or some other legume as a cover crop is not common in BH, but is sometimes practiced by farmers who do not have access to manure (which was the case on our field) or want to decrease the production costs with the application of green manures. Even though we did not measure mineral N in the first year, it is obvious from the N_2_O emissions that ploughing the cover crop in autumn prior to the first experimental year and rototilling it in spring released more C and N than in RT and NT, likely because alfalfa residues had a longer contact time with the soil. A similar finding was reported by [54] for a Mediterranean cover crop system. Secondly, 2014 was an extraordinary wet year (Fig 1), with WFPS values around 70–80% in CT and exceeding 90% in NT in the period after fertilization (Fig 2). It is well known that N_2_O emissions from denitrification are greatest at WFPS values around 80%, (e.g. [55]), whereas larger WFPS values favour reduction of N_2_O to N_2_ [56]. For instance, larger N_2_O emissions at 80% compared to 100% WFPS have been reported by Ciarlo et al. [57], who observed decreasing N_2_O emission in saturated soil. In the wet summer of 2014, we observed several occasions of waterlogging during sampling in NT plots. RT plots had WFPS values intermediate to RT and NT, which resulted in intermediate post-fertilization N_2_O emissions. In contrast, the second year was quite dry (Fig 1), and the differences in WFPS between treatments were less pronounced. Post-fertilization emissions occurred during a period with decreasing WFPS, and were largest in RT. We have no explanation for this finding other than that NH_4_+ and NO_3_^-^ values were largest in RT during this period, pointing at enhanced mineralisation and nitrification in this treatment at 60–80% WFPS.

### Soil properties

Our experiment was conducted on a clayey loam low in SOC (1.34%). The soil had been under intensive arable cropping for the past decade with limited application of manure. Arable cultivation without manures decreases the SOM content over time and consequently weakens the structural stability of the soil [58]. Twenty months into our experimental trial, NT soil had the largest bulk density and may have experienced soil compaction [59], which is known to be a problem in soils with low aggregate stability [60]. Negative effects of soil compaction on crop yields are well documented [61]. In our study, NT soil had 8.2% less total porosity than CT soil and smaller proportion of meso and macro pores (Table 1). This led to reduced water infiltration and occasional waterlogging during more intense and prolonged rainfall events, especially in 2014. In both years, large rainfalls after sowing led to soil crusting, even in the presence of mulched crop residues in RT and NT, delaying the emergence of seedlings relative to CT. This effect was especially pronounced in NT, which led to significant reduction (approx. 25%) in the abundance of corn plants relative to CT and RT in 2014. Another notable observation was that both corn and barley plants in CT and RT were taller irrespective of their phenological stage and that the phenological development was 10–15 days ahead this of NT. Among the positive effects of NT and RT in 2015 was a larger proportion of soil micro- and macro-pores, respectively (Table 1), resulting in larger soil water retention during drought periods, which probably contributed to equal (NT) or larger (RT) barley yields compared with CT in 2015, despite later phenological development and general crop failure.

### N_2_O emission factor and intensity

Assuming a background emission of 1 kg N_2_O-N ha^-1^ y^-1^ [37], we obtained mean N_2_O emission factors (EF) ranging from 2.7 to 4.8% of fertilizer N applied. Since these emission factors are markedly larger than the IPCC Tier 1 EF (1% of applied fertilizer N), we tried to estimate the additional N input from alfalfa residues during the first year (Fig 4), based on the results from Kelner et al. [39] and adjusted for alfalfa yields from our study field [40]. Adding this amount of N to the fertilizer N, reduced N_2_O emission factors in 2014 to 1.5, 1.6 and 2.7% for NT, RT and CT respectively, which is closer, but still above the IPCC Tier 1 EF of 1% [37]. In 2015, emission factors were 0.7, 1.0 and 1.5% for NT, CT and RT respectively, which is close to the IPCC Tier 1 EF, despite the general crop failure in this year. Given the uncertain estimate of extra N input in 2014, and the insignificant differences in the EF across tillage treatments in 2015, we conclude that tillage regime had no measurable effect on the N_2_O emission factors in our experiment.

To assess the N_2_O footprint of cropping methods, yield-scaled approaches are increasing employed [38, 62]. Calculated as N_2_O intensity (g N_2_O-N Mg grain^-1^), RT had the lowest intensity in both years. We used the results from 2014 for further comparison, since the high values in 2015 essentially reflected crop failure. Yield-scaled N_2_O emissions in 2014 ranged from 83.2 (RT) to 161.7 g N_2_O-N Mg^-1^ (NT) and this difference was statistically significant. These results are within range of 77.1–391.8 g N_2_O-N Mg^−1^ as reported by Guo et al. [63], but larger than those reported by Halvorson et al. [64] (31–67 g N_2_O-N Mg^−1^) and Venterea et al. [65] (46–100 g N_2_O-N Mg^−1^) for the same crop (corn). Our intensities are smaller than those reported by Burzaco et al. [66] for similar fertilization rates (211–285 g N_2_O-N Mg^−1^) and markedly smaller than the 1.3–2.0 kg N_2_O-N Mg^−1^ reported by Gagnon et al. [67].

### Environmental vs. economic benefits

It has been widely recognised that conservation tillage practices have a beneficial effect on soil properties in cropping systems compared to conventional practices [68]. However, the adoption of NT in Europe is still limited in comparison with the Americas, despite extensive research in this area [69]. The relative advantages of NT and CT depend on a number of aspects, grouped roughly into agronomic and environmental factors [70]. According to Soane et al. [69], the opinions and choices of farmers related to tillage will be dictated primarily by agronomic factors, whereas environmental factors are of a more general concern for soil and landscape protection and climate change. While the agronomic benefits of alternative tillage methods are easy to recognize, the likelihood for their adoption is constrained due to relative uncertainty about the economic benefits [71, 72].

Summarizing stipulated costs and revenues over the two cropping seasons, including one year of crop failure, it is notable that RT had smaller yield-scaled N_2_O emissions than CT and NT, while generating the largest net returns per ha. These findings are in agreement with and support the current situation in Europe, where intermediate forms of tillage have been adopted much more widely than NT [69]. While only a small reduction of variable costs in NT could be achieved in our experiment due to the increased costs for weed control, the main reason for small net returns and large yield-scaled N_2_O emissions in NT were lower crop yields compared to CT and RT. Conservation tillage initially leads to yield reduction of varying degree depending on crop type, tillage, soil properties, climate and crop rotation [73, 74]. However, it is expected that the yield gap between reduced and conventional tillage will level out over time, as the soil structure, water infiltration and root growth improve in the surface soil under continuous NT systems [75].

Based on our findings and the current socio-economic conditions in BH, we conclude that a wider acceptance of NT is not realistic at the moment, especially among risk-averse smallholder farmers who lack knowledge and financial capital. However, our result also show that RT could be a compromise between CT and NT, given the current conditions, because it can be both N efficient and economically acceptable. RT appeared to be more resilient to weather extremes, particularly during the dry year of 2015. Our study was conducted in “continental” BH, characteristic for the northern lowlands and river valleys of central, eastern and western BH, which is central for BH’s intensive agricultural production of a wide range of crops. Yet, further research is needed in other agroclimatic zones and soil types of BH to assess tillage effects at the national level. In addition, longer-term studies are needed to make more reliable projections of yield levels and environmental savings.

## Conclusion

Fertilization was found to be the main driver of N_2_O emissions irrespective of tillage treatment. However, clear treatment effects outside the period directly affected by fertilization were noted, indicating the importance of crop residue management and tillage on soil structure, temperature and moisture. Annual emissions were different between tillage treatments, but this depended on the year. NT had the smallest N_2_O emission in both years, while CT had the largest emission in the first year and RT the largest emission in the second year. When normalized for yield, RT had the smallest N_2_O intensity in both years. The emission factors were within or slightly above the uncertainty range of the IPCC Tier 1 EF. Reduction in variable costs associated with NT was small due to the increased costs of weed control. Moreover, low yields in NT in both years resulted in economically inacceptable returns. On the other hand, the reduced number of operations in case of RT generated higher net returns due to reduced production costs. Based on our results, we conclude that RT could be a feasible way to improve N yields and net returns, while reducing yield-scaled N_2_O emissions under the given agro-ecological and socio-economic conditions of BH. Future studies with the objective to mitigate N_2_O and other greenhouse gas emissions, improve crop yields and N use efficiency from agricultural systems in the region are needed, including long-term observation, that can integrate over the large interannual weather variability in central BH. Studies on the effect of CA principles other than reduced tillage (e.g. cover crops, crop rotation) are needed if region-specific production system are to be designed to optimize crop production both environmentally and economically.
